# Clinical-Radiomics Signature Predicts Pathologic Complete Response After Neoadjuvant Therapy in Oesophageal Squamous Cell Carcinoma

**DOI:** 10.1093/icvts/ivag024

**Published:** 2026-02-04

**Authors:** Liqiang Shi, Xipeng Wang, Xueyu Chen, Yuqin Cao, Chengqiang Li, Yaya Bai, Zenghui Cheng, Dong Dong, Xiaoyan Chen, Yajie Zhang, Hecheng Li

**Affiliations:** Department of Thoracic Surgery, Ruijin Hospital, Shanghai Jiao Tong University School of Medicine, Shanghai 200025, China; Department of Thoracic Surgery, Ruijin Hospital, Shanghai Jiao Tong University School of Medicine, Shanghai 200025, China; Department of Thoracic Surgery, Ruijin Hospital, Shanghai Jiao Tong University School of Medicine, Shanghai 200025, China; Department of Thoracic Surgery, Ruijin Hospital, Shanghai Jiao Tong University School of Medicine, Shanghai 200025, China; Department of Thoracic Surgery, Ruijin Hospital, Shanghai Jiao Tong University School of Medicine, Shanghai 200025, China; Department of Nuclear Medicine, Ruijin Hospital, Shanghai Jiao Tong University School of Medicine, Shanghai 200025, China; Department of Radiology, Ruijin Hospital, Shanghai Jiao Tong University School of Medicine, Shanghai 200025, China; Department of Thoracic Surgery, Ruijin Hospital, Shanghai Jiao Tong University School of Medicine, Shanghai 200025, China; Department of Pathology, Ruijin Hospital, Shanghai Jiao Tong University School of Medicine, Shanghai 200025, China; Department of Thoracic Surgery, Ruijin Hospital, Shanghai Jiao Tong University School of Medicine, Shanghai 200025, China; Department of Thoracic Surgery, Ruijin Hospital, Shanghai Jiao Tong University School of Medicine, Shanghai 200025, China

**Keywords:** oesophageal squamous cell carcinoma, radiomics, active surveillance, pathologic complete response, neoadjuvant therapy

## Abstract

**Objectives:**

Neoadjuvant therapy (NAT) significantly improves the pathologic complete response (pCR) rates in patients with locally advanced esophageal squamous cell carcinoma (ESCC). Emerging evidence suggests that patients with pCR may experience favourable outcomes and could be considered for active surveillance strategies to delay surgery. This study aims to develop a clinical-radiomics model to predict pCR after NAT in ESCC.

**Methods:**

We retrospectively enrolled 236 patients with locally advanced ESCC who received NAT at our centre and randomly assigned them to training and test cohorts (3:2 ratio). Radiomics features were extracted from tumour regions segmented on post-NAT contrast-enhanced computed tomography (CT) scans. After feature selection, a predictive model integrating radiomics and clinical variables was developed using logistic regression and visualized as a nomogram. Model performance was evaluated using area under the curve (AUC), accuracy, sensitivity, and specificity.

**Results:**

The clinical-radiomics model achieved an AUC of 0.91 (95% confidence interval [CI]: 0.86-0.95), accuracy of 0.84, sensitivity of 0.89, and specificity of 0.81 in the training cohort, and an AUC of 0.84 (95% CI: 0.76-0.92), accuracy of 0.78, sensitivity of 0.84, and specificity of 0.74 in the test cohort. Calibration curves demonstrated good agreement between predicted and observed outcomes, and decision curve analysis confirmed the model’s clinical utility.

**Conclusions:**

The clinical-radiomics model accurately predicts pCR following NAT in ESCC and may guide personalized treatment strategies.

## Graphical abstract

**Figure:**
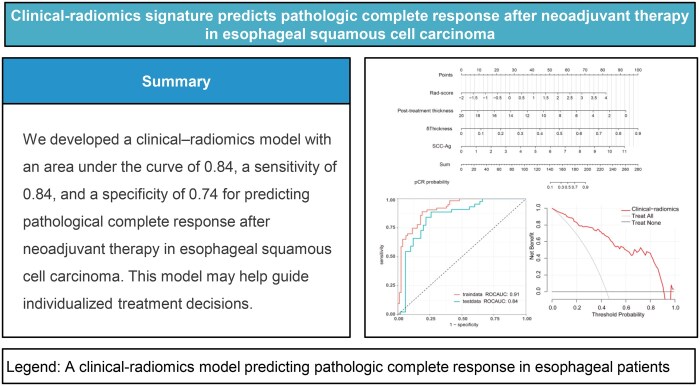

## INTRODUCTION

Oesophageal cancer is the seventh most common cancer and sixth leading cause of cancer-related death worldwide,^1^ with China accounting for over half of global ESCC cases.^2^ Neoadjuvant chemoradiotherapy (NCRT) followed by surgery represents the standard treatment for locally advanced ESCC.^3^^,^^4^ The emergence of immune checkpoint inhibitors has revolutionized ESCC therapy. Recent clinical trials, including PALACE-2,^5^ NCT04437212,^6^ and NEOCRTEC1901,^7^ showed that neoadjuvant immunotherapy plus chemoradiotherapy (NICRT) is emerging as first-line treatment for locally advanced ESCC.

Neoadjuvant therapy (NAT) provides high pathologic complete response (pCR) rates (43.2%-55.6%),^4^^,^^6–8^ supporting the rationale for exploring active surveillance approaches. Active surveillance is a nonoperative approach in which patients predicted to have achieved pCR after NAT undergo close monitoring instead of immediate surgery. Research shows it provides similar disease control and survival rates as oesophagectomy after post-NCRT.^9^ Recently, the SANO trial reported that overall survival (OS) at 2 years following active surveillance was noninferior to that of standard surgery, further supporting its potential as an alternative to surgery after NAT. The success of this strategy relies on accurately predicting pCR after neoadjuvant treatment to identify patients who can benefit from organ preservation and ensure effective treatment for those with residual disease. Thus, predicting pCR is crucial for the personalized treatment of ESCC.

Previous studies have investigated the prediction of pCR in ESCC through traditional evaluation techniques such as computed tomography (CT), magnetic resonance imaging (MRI), positron emission tomography (PET)/CT, or endoscopic biopsy. However, the effectiveness of these approaches seems to be insufficiently accurate to identify complete responders,^10^ and some residual disease after NCRT was not detected by endoscopic biopsies.^11^ Therefore, there is an urgent need for novel diagnostic methods to identify pCR patients.

Radiomics enables high-throughput extraction of quantitative imaging features and has shown promise for predicting treatment response in various cancers.^12^^,^^13^ However, few studies have applied post-treatment radiomics to predict pCR following NAT in ESCC. Here, we evaluated the predictive value of CT-derived radiomics from post-NAT imaging in patients with locally advanced ESCC.

## MATERIALS AND METHODS

### Ethical statement

This retrospective study was approved by the institutional review board of Ruijin hospital and was exempt from the requirement for written informed consent. The study was conducted in accordance with the Declaration of Helsinki. Collection and storage of data from included patients is consistent with requirements outlined in the WMA Declaration of Taipei.

### Patients and clinical characteristics

The patient enrolment flowchart is described in **Figure S1**. A total of 236 patients with locally advanced ESCC in our hospital from January 2016 to December 2024 were enrolled. The inclusion and exclusion criteria of patients were listed in **eAppendix 1**. The treatment scheme of NCRT/NICRT was described in **eAppendix 2.** Clinical characteristics after NAT were collected. In addition, the maximum oesophageal wall thickness before treatment and the corresponding thickness after treatment were measured on CT images. The percentage change in thickness (δThickness%) was calculated as the reduction in maximum tumour thickness after NAT divided by the baseline maximum tumour thickness from the pretreatment scans.^14^

Patients were classified into pCR and non-pCR groups based on postoperative pathology. The pCR was defined as ypT0N0, indicating no viable tumour cells in surgical specimens (primary tumour and lymph nodes) following NCRT/NICRT.^8^^,^^13^^,^^14^ The cohort was randomly divided into training and test sets (3:2 ratio).

### CT radiomics feature extraction

**Figure 1.**
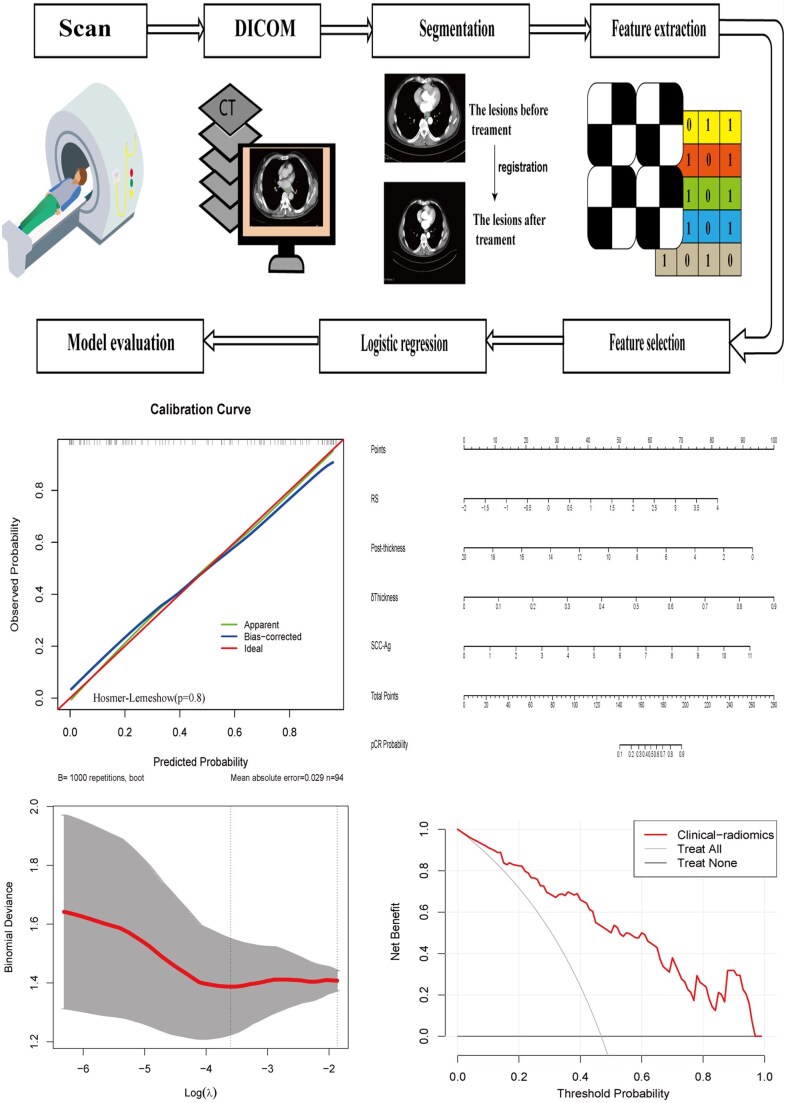
The Flowchart of Predictive Model Building

The flowchart of predictive model building is illustrated in **Figure 1**. Contrast-enhanced CT scans were collected before NAT and at 4 weeks after its completion. A thoracic surgeon delineated 3-dimensional regions of interest (ROI) encompassed the whole tumour using 3D slicer (version, 5.2.2, Stable Release, https://www.slicer.org/), which was then verified and confirmed by 2 radiologists. All operators were blinded to patients’ clinical and histopathological information. The pretreatment ROI was drawn along the contour of the primary oesophageal tumour. The post-treatment CT images of each patient were then registered with the corresponding pretreatment images. Next, the contour of the pretreatment ROI was projected onto the post-treatment images. The post-treatment ROI was manually adjusted from the pretreatment ROI to compensate for the surrounding tumour shrinkage after treatment and to maintain the consistency of the cranial-to-caudal anatomical range.^15^^,^^16^ The radiomic features were extracted with a resample filter and voxel resampling size set at 1 × 1 × 5 m^3^.

A total of 1316 image features from post-treatment CT images were extracted. The features were divided into 3 categories: original, wavelet-filtered, and Laplacian of Gaussian–filtered images (1-5 mm, 1 mm interval).^13^ ComBat correction was applied to harmonize batch effects (**eAppendix 3**).

### Feature selection

Radiomics features were normalized using *z*-score transformation. Feature selection in the training cohort proceeded through 3 steps: (1) removing one feature from pairs with correlation coefficients >0.95; (2) excluding features with *P* > .05 in univariate analysis between pCR and non-pCR groups; and (3) identifying the top 15 features using support vector machine-recursive feature elimination.^13^^,^^17^ Following this, the most significant features were selected by the least absolute shrinkage and selection operator (LASSO) with 5-fold cross-validation method.

The significant radiomics features were weighted with their coefficients and summed to calculate the Rad-score (Rad-score = coefficient 1 × feature 1+ coefficient 2 × feature 2…).^14^ Regarding clinical features, the backward elimination was used to select significant clinical factors. We combined Rad-score and clinical features to construct a clinical-radiomics model to explore whether the combined model outperforms the individual model.

### Model construction

Radiomics and clinical-radiomics models were developed using logistic regression. Model performance was assessed using receiver operating characteristic (ROC) curves, with area under the curve (AUC), accuracy, sensitivity, specificity, positive predictive value (PPV), and negative predictive value (NPV) calculated. The optimal model was visualized as a nomogram, with calibration assessed using calibration curves and the Hosmer–Lemeshow test. Decision curve analysis (DCA) evaluated clinical net benefit.

### Statistical analysis

Statistical analyses were performed using R software. Categorical variables were compared using χ^2^ tests, and continuous variables using independent-samples *t* tests or Mann–Whitney *U* tests, as appropriate.^13^^,^^14^ Correlations between variables were assessed using Pearson or Spearman analysis. The AUC values were compared using the DeLong test. The impact of NICRT on pCR was evaluated using binary logistic regression. Statistical significance was set at 2-sided *P* < .05.

## RESULTS

### Patients’ characteristics

A total of 236 patients were enrolled, comprising 129 (54.7%) with non-pCR and 107 (45.3%) with pCR. A total of 101 patients received NICRT (45.5% pCR), and 135 patients received NCRT (45.1% pCR). Binary logistic regression showed that, compared with NCRT, NICRT did not significantly affect the rate of pCR (hazard ratio [HR] = 0.977, 95% confidence interval [CI] = 0.58-1.64, *P* = .93). As shown in **Table 1**, the δThickness% in pCR patients was significantly higher than that in non-pCR patients, and the post-treatment oesophageal wall thickness in pCR patients was significantly lower than that in non-pCR patients. Other baseline characteristics between the 2 groups were no significant differences. The training cohort comprised 79 non-pCR and 63 pCR patients, while the test cohort comprised 50 and 44, respectively.

**Table 1.** Baseline Demographic and Clinical Characteristics of Patients

Characteristics	Patients			
	Total (*n* = 236)	Non-pCR (*n* = 129)	pCR (*n* = 107)	*P* value
Age, mean (SD), years	64.1 (6.9)	64.3 (6.7)	63.8 (7.0)	.62
BMI (kg/m2), mean (SD)	22.6 (2.94)	22.4 (3.0)	22.8 (2.9)	.30
Gender				
Male	201	111	90	.67
Female	35	18	17	
Clinical T stage^a^				
T2	23	10	13	.49
T3	198	110	88	
T4	15	9	6	
Clinical N status				
N-	55	36	19	.06
N+	181	93	88	
Clinical stage				
II	68	40	28	.61
III	153	80	73	
IV	15	9	6	
Location				
Upper	32	14	18	.40
Middle	125	71	54	
Lower	79	44	35	
Tumour length (cm), mean (SD)				
	4.1 (1.8)	4.5 (2.1)	4.3 (1.9)	.37
Posttreatment				
Leucocyte (10^9^/L), mean (SD)	4.8 (2.1)	4.9 (2.2)	4.6 (1.9)	.60
Albumin(g/L), mean (SD)	37.3 (3.7)	37.3(4.1)	37.5 (3.2)	.93
Hemoglobin(g/L), mean (SD)	118.3 (14.5)	119.53 (14.63)	116.96 (14.34)	.17
Neutrophil (10^9^/L), mean (SD)	3.2 (1.9)	3.3 (2.1)	3.0 (1.7)	.48
Lymphocyte (10^9^/L), mean (SD)	1.0 (1.3)	1.1 (1.8)	0.9 (0.4)	.63
Platelet (10^9^/L), mean (SD)	183.7 (62.4)	183.4 (59.8)	183.9 (65.6)	.80
AFP (μg/L)	3.3 (1.6)	3.3 (1.5)	3.3 (1.7)	.53
CA125 (U/mL)	11.1 (6.6)	11.4 (7.16)	10.7 (5.8)	.79
CA199 (U/mL)	10.9 (9.8)	10.9 (10.3)	10.8 (9.2)	.81
CEA (ng/mL), mean (SD)	2.1 (1.3)	2.2(1.2)	2.0 (1.4)	.08
SCC-Ag (ng/mL), mean (SD)	1.2 (1.6)	1.2 (1.7)	1.1 (1.5)	.98
CT measurement				
Pre-thickness by CT (mm), mean (SD)^a^	14.0 (5.4)	13.4 (5.0)	14.6 (5.6)	.11
Post-thickness by CT (mm), mean (SD)^b^	6.3 (6.9)	7.6 (9.0)	4.7 (1.6)	<.001
δThickness% by CT (%), mean (SD)^c^	52.9 (31.7)	42.3 (38.7)	65.6 (10.6)	<.001

Abbreviations: AFP, alpha-fetoprotein; BMI, body mass index; CA199, carbohydrate antigen 199; CA125, carbohydrate antigen 125; CEA, carcinoembryonic antigen; pCR, pathologic complete response; SCC-Ag, squamous cell carcinoma antigen.

a Pre-thickness was defined as the maximum thickness on CT before treatment.

b The slice of post-thickness (after treatment) was corresponding to the slice of pre-thickness on CT.

c δThickness was calculated as the reduction in maximum tumour thickness after NAT divided by the baseline maximum tumour thickness from the pretreatment scans.

### Model construction

**Figure 2.**
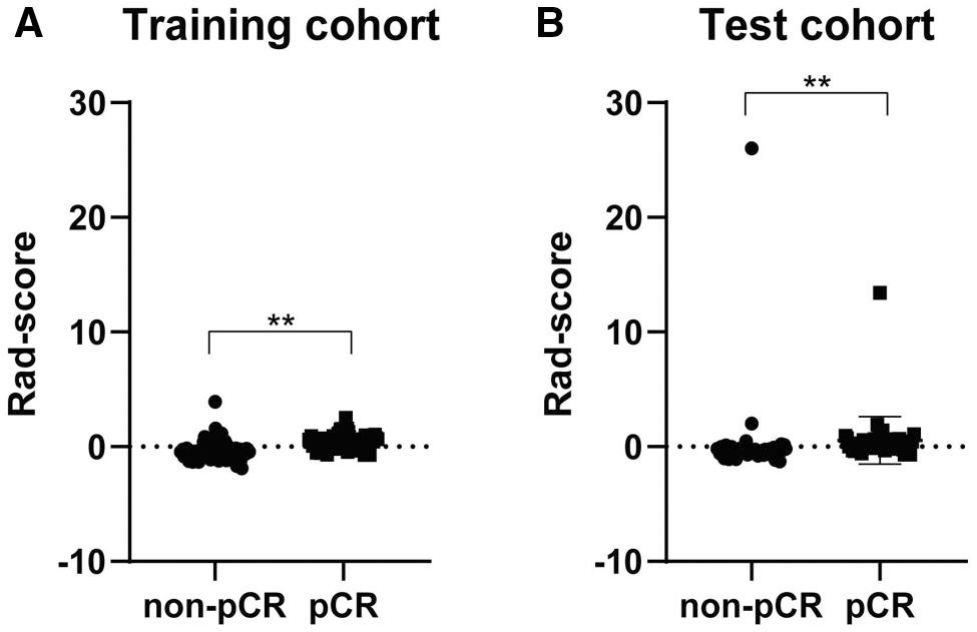
Comparison of Rad-Score in 2 Groups (pCR vs non-pCR). (A) Rad-Score in the Training Cohort. (B) Rad-Score in the Test Cohort. **P < .01. Abbreviation: pCR, pathologic complete response

For the radiomics models, the selected features were described in **Table 2**. As for the clinical model, the selected features were δThickness%, post-treatment oesophageal wall thickness, and squamous cell carcinoma antigen (SCC-Ag). The Rad-score was also calculated. As shown in **Figure 2**, the Rad-score of non-pCR (median: −0.45) was significantly lower than that of pCR (median: 0.49) in the training cohort. Besides, the Rad-score of non-pCR (median: −0.50) was also significantly lower than that of pCR (median: 0.11) in the test cohort. The result showed that the Rad-score may be a discriminative factor for identifying non-pCR and pCR. Then, the Rad-score and clinical features were combined to construct a clinical-radiomics model. The radiomics and clinical-radiomics models were constructed by logistic regression.

**Table 2.** Selected Features of the Radiomics Model

Models	Image filter^a^	Feature group	Feature name	Coefficients
Radiomics model	Wavelet (LHH)	GLCM	ClusterProminence	0.625
	Wavelet (LHH)	GLCM	ClusterShade	−0.319
	Wavelet (HLH)	GLCM	ClusterProminence	−0.297
	Wavelet (HLH)	GLCM	MCC	0.282
	Wavelet (HLL)	firstorder	Maximum	0.228
	Wavelet (LHH)	GLSZM	LargeAreaHighGrayLevelEmphasis	−0.201

Abbreviations: GLCM, Gray Level Co-occurrence Matrix; GLSZM: Gray Level Size Zone Matrix.

a For wavelet filtration, “H” and “L” represent high pass filter and low pass filter in the x, y, z direction.

### Model validation and comparison

The clinical-radiomics model demonstrated optimal performance (**Table 3**), achieving an AUC of 0.91 (95% CI: 0.86-0.95), accuracy of 0.84, sensitivity of 0.89, specificity of 0.81, NPV of 0.90, and PPV of 0.79 in the training cohort, and an AUC of 0.84 (95% CI: 0.76-0.92), accuracy of 0.78, sensitivity of 0.84, specificity of 0.74, NPV of 0.84, and PPV of 0.74 in the test cohort.

**Table 3.** Performance of Predictive Models for Predicting pCR in the Training and Test Cohorts

Cohort	Models	Accuracy	Sensitivity	Specificity	PPV	NPV	AUC (95% CI)
Training	Radiomics	0.77	0.75	0.79	0.75	0.79	0.83 (0.76-0.89)
	Clinical-radiomics	0.84	0.89	0.81	0.79	0.90	0.91 (0.86-0.95)
Test	Radiomics	0.75	0.57	0.90	0.83	0.70	0.81 (0.72-0.90)
	Clinical-radiomics	0.78	0.84	0.74	0.74	0.84	0.84 (0.76-0.92)

Abbreviations: AUC, area under the curve; CI, confidence interval; pCR, pathological complete response; PPV, positive predictive value; NPV, negative predictive value.

**Figure 3.**
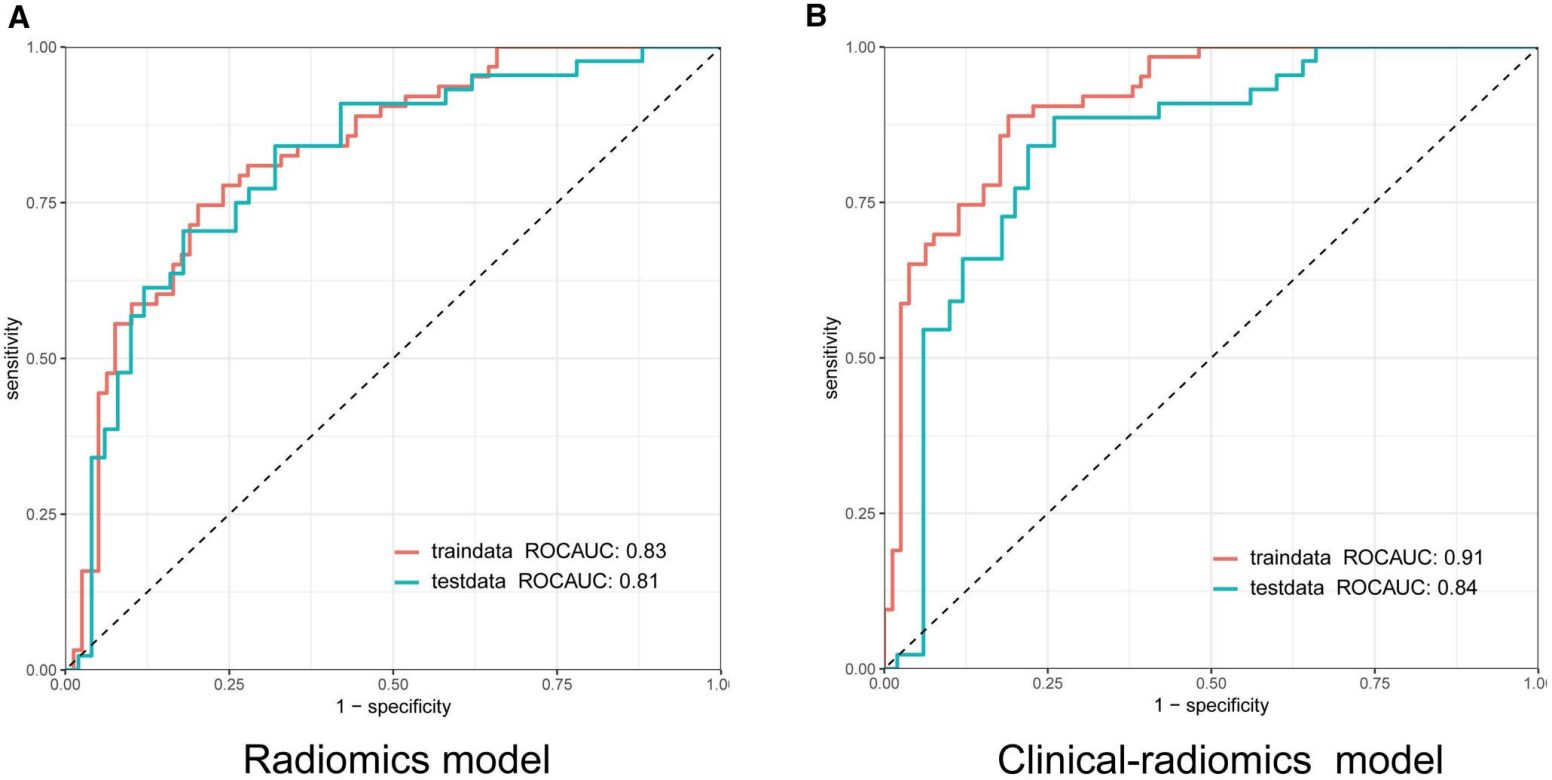
The ROC of the 2 Models. (A) Radiomics Model. (B) Clinical-Radiomics Model. Abbreviations: ROC, receiver operating characteristic; AUC, area under the curve

The ROC curves showed that the clinical-radiomics model exhibited the best predictive efficiency (**Figure 3**). The clinical-radiomics model significantly outperformed the radiomics model in AUC in the training cohort, although this difference did not reach statistical significance in the test cohort (**Figure S2**).

These findings indicate that the clinical-radiomics model is optimal for predicting treatment response following NAT.

### Nomogram construction

**Figure 4.**
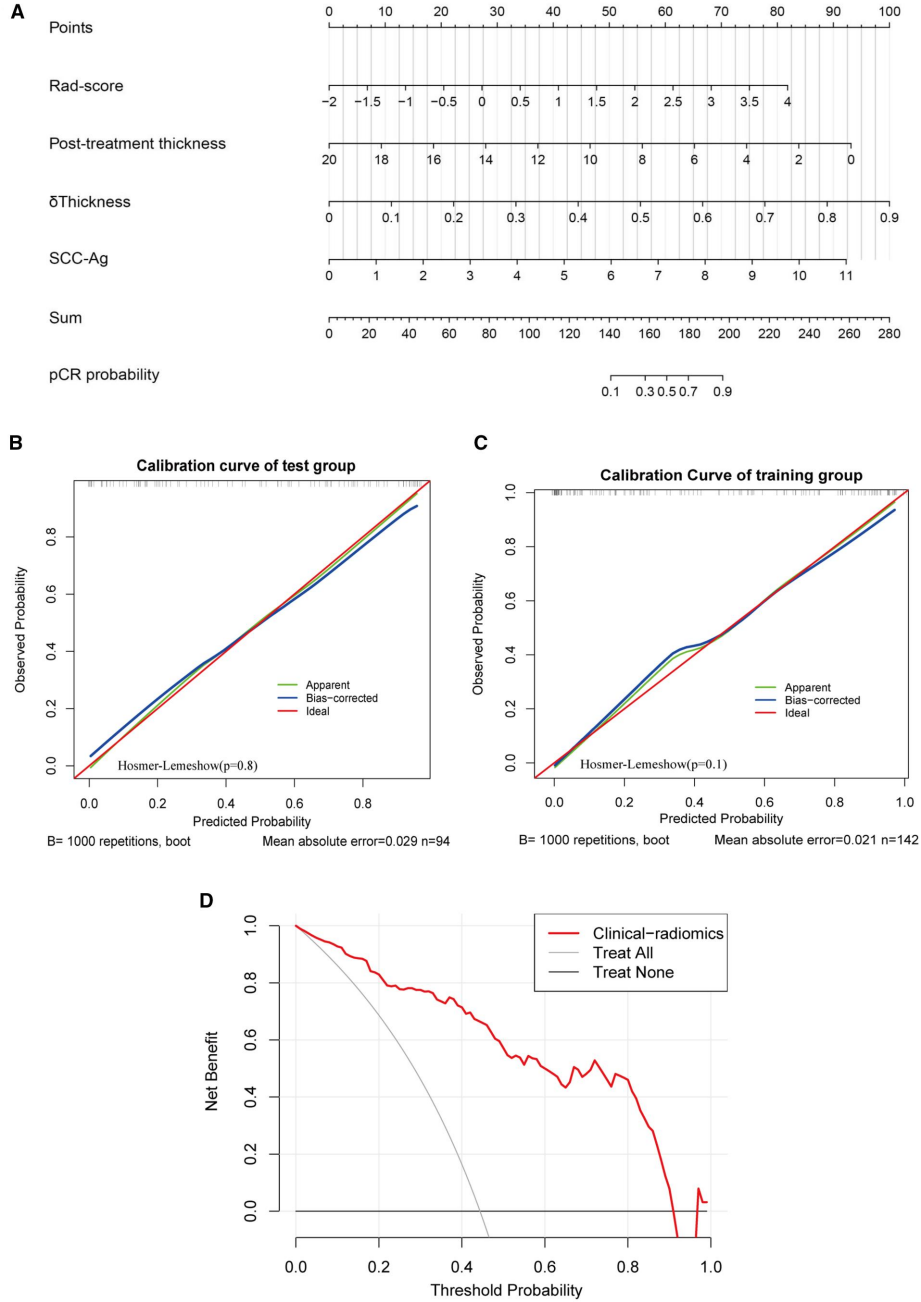
Nomogram, Calibration Curve, and Decision Curve Analysis of Clinical-Radiomics Model. (A) Nomogram of the Clinical-Radiomics Model. (B) Calibration Curves in the Training Cohort. (C) Calibration Curves in the Test Cohort. (D) Decision Curve Analysis of the Clinical-Radiomics Model. Abbreviation: pCR, pathologic complete response

The clinical-radiomics model incorporating Rad-score and clinical factors was established and presented with a nomogram (**Figure 4A**). The calibration curve of the nomogram for the treatment response demonstrated good agreement between nomogram prediction and actual observation (**Figure 4B and C**). The Hosmer-Lemeshow test showed no statistical significance in the training set or validation set (*P* values of .1 and .8), indicating that the nomogram had a good fit. The decision curve analysis for the nomogram is shown in **Figure 4D**. The DCA demonstrated that when the threshold probability was between 3% and 90%, the nomogram may be more advantageous than the treat-all or treat-none strategy.

These findings demonstrate that the clinical-radiomics model performs well in clinical practice.

## DISCUSSION

Organ-preserving strategies have been proposed in other solid tumours, such as rectal cancer and penile cancer.^18–20^ Specifically, this organ-preserving strategy for rectal cancer following NAT achieves good outcomes.^19^^,^^20^ The benefit of standard oesophagectomy after NAT for clinically complete response (cCR) remains controversial. In the (pre)SANO clinical trial, van der Wilk found no significant differences in OS and progression-free survival between patients with cCR who underwent active surveillance and those who received immediate surgery. Furthermore, active surveillance followed by delayed surgery for recurrent disease was not associated with a higher rate of distant dissemination or more severe adverse postoperative outcomes.^9^ Van der Wilk reported the results of a phase III clinical trial demonstrating that, among oesophageal cancer patients who achieved cCR following neoadjuvant therapy (NAT), active surveillance strategy resulted in a comparable 2-year OS with that of standard surgical treatment.^21^ This supports the rationale for developing accurate prediction models to identify potential pCR patients who may benefit from organ-preserving strategies.

The success of oesophageal preservation depends on the prediction of pCR after NAT. Endoscopic biopsy is the main method for assessing response, but some residual disease may be missed.^11^ As pCR can only be definitively confirmed through histological examination, establishing a noninvasive approach to assess response after NAT is crucial for developing personalized strategies for locally advanced ESCC.

In recent years, noninvasive radiomics has shown promise in predicting treatment response and prognostic outcomes.^12^^,^^13^^,^^22^ The underlying principle is that the genetic heterogeneity of tumours is translated into histopathological features, which can be captured through medical imaging.^23^ Some study found that the fluorodeoxyglucose (FDG)-PET/CT radiomics can effectively predict pCR in oesophageal cancer (AUC: 0.77-0.81).^24^^,^^25^ However, these studies primarily focused on oesophageal adenocarcinoma. Compared with PET-based radiomics model, the sensitivities of FDG-PET/CT scans and CT scans are comparable.^26^ Some researchers used the pretreatment CT radiomics to predict pCR in ESCC receiving NAT with good performance (AUC: 0.80-0.85).^13^^,^^27^ Nevertheless, these models are primarily aimed to identify which patients can benefit from NAT, rather than making judgments after NAT. Thus, we aimed to explore the predictive value of clinical-radiomics for predicting pCR in ESCC after NAT.

In our study, Gray-level co-occurrence matrix (GLCM) parameters predominated among the selected features. Previous studies have demonstrated that GLCM serves as a prognostic indicator for OS in pancreatic cancer^28^ and small‐cell lung cancer,^29^ and may function as a biomarker for malignancy stratification in colorectal cancer.^30^ Additionally, GLCM parameters have been used to differentiate pCR from non-pCR.^31^ In our study, the Rad-score was significantly higher in the pCR group than in the non-pCR group across both training and test cohorts. These findings suggest that radiomics features may be associated with treatment response.

We developed a clinical-radiomics model for predicting pCR to NAT in locally advanced ESCC. The model was internally validated with good performance, achieving an AUC of 0.92 in the training cohort and 0.84 in the test cohort. Then, the calibration curve and DCA further validated its clinical utility. The model demonstrated promising potential in predicting treatment response in ESCC patients who received NAT, facilitating personalized strategies. Patients predicted to be non-pCR would undergo standard oesophagectomy, whereas those identified as pCR could be considered for active surveillance. The DCA indicated that the model provided a net benefit across a threshold probability range of 3% to 90%. For example, at a threshold probability of 80%, the model could reduce the number of esophagectomies by 20% without increasing misclassified non-pCR patients. The model’s ability to accurately predict pCR enabled the avoidance of surgery in 44 of 220 patients, indicating that 40% of the 107 true pCR patients could potentially benefit from active surveillance. Threshold selection should balance clinical benefit and misclassification risk and be tailored to patient preferences. The model exhibited good performance to guide active surveillance. Besides, the model should complement existing diagnostic tools. Integration with endoscopic biopsy and PET/CT analysis can further enhance organ preservation after NAT.

The clinical-radiomics model was constructed using Rad-score, δThickness%, post-treatment oesophageal wall thickness, and SCC-Ag. Rad-score has been considered a discriminative factor for identifying non-pCR and pCR in ESCC receiving neoadjuvant treatment.^27^^,^^32^ SCC-Ag is a subfraction of the tumour antigen TA-4, a 48-kDa glycoprotein first isolated by Kato and Torigoe.^33^ SCC-Ag has been one of the most commonly used in the diagnosis of a variety of malignant tumour to date, and it may be involved in the malignant behaviour of squamous cell cancers, functioning in invasion and/or metastasis.^33^^,^^34^ Recent research also indicates that overexpression of SCC-Ag is a significant factor in drug resistance in oesophageal cancer.^35^ Moreover, SCC-Ag levels were predictive for advanced tumour stage, recurrence, and survival of patients with ESCC.^36^ Oesophageal wall thickness was also important predictive factors. Li found that most of the locally advanced ESCC with pretreatment maximal oesophageal wall thickness ≥20 mm did not achieve pCR after chemoradiotherapy.^37^ However, evaluation of the therapeutic response and prognosis solely by pre- or post-chemoradiotherapy maximal oesophageal wall thickness is often influenced by individualized differences.^38^ In contrast, δThickness% is less influenced by patient characteristics, and thus it may have more prognostic value.^38^ The δThickness% is independently associated with pCR and recurrence among ESCC patients who undergo NAT and surgery.^38^ In our study, pCR patients exhibited significantly higher δThickness% and lower post-treatment oesophageal wall thickness compared with non-pCR patients. These results were consistent with previous findings regarding tumour thickness–derived parameters in pCR patients receiving NAT.^32^ The δThickness% may be associated with pCR. Integrating clinical variables with radiomics achieved an AUC of 0.84, suggesting that such combination enhances predictive performance.

Our study has several limitations. First, this single-centre retrospective study had a limited sample size; external validation using large-scale multicentre cohorts is warranted. Second, although ComBat correction was applied to CT scans, some variability may persist. Future multicentre studies should implement standardized imaging protocols and unified reconstruction and quality control procedures to minimize inter-scanner variability. Finally, our model relied solely on CT imaging. Given that liquid biopsy noninvasively captures tumour-derived biomarkers and PET/CT is established for staging and response assessment in ESCC, a multimodal approach integrating these modalities with CT radiomics may improve pCR prediction and merits future investigation.

## CONCLUSION

In conclusion, the clinical-radiomics model effectively predicts treatment response following NAT and may guide personalized treatment decisions.

## Supplementary Material

ivag024_Supplementary_Data

## Data Availability

The datasets generated during and analysed during the current study are available from the corresponding author on reasonable request.
