# Supplementary material for: Clinical-Radiomics Signature Predicts Pathologic Complete Response After Neoadjuvant Therapy in Oesophageal Squamous Cell Carcinoma
Source: Interdiscip Cardiovasc Thorac Surg. 2026 Feb 4;41(2):ivag024. doi: 10.1093/icvts/ivag024 (PMC12881974; doi:10.1093/icvts/ivag024)

Supplementary Materials

eAppendix 1. **The inclusion and exclusion criteria of patients**

Patients with locally advanced ESCC (T_2-4a_N_any_M_0_) were included in this study. Eligible patients were 18-75 years old, had Eastern Cooperative Oncology Group performance status score of 0-1. The key exclusion criteria were autoimmune disease, ongoing immunosuppressive therapy and having received chemotherapy, radiotherapy, target therapy or immune therapy for this or any other cancers[1].

eAppendix 2. **Treatment schemes for NCRT and NICRT**

Patients received NCRT before surgery, consisting of carboplatin (area under the curve of 2 mg/mL/min) and paclitaxel (50 mg/m2) once a week for 5 weeks, radiotherapy (23 fractions of 1.8 Gy, 5 fraction per week) [CROSS regimen][2]. In the NICRT regimen, anti-PD-1 inhibitor pembrolizumab (2 mg/kg) is administered on days 1 and 22 in addition to NCRT [3]. Radical esophagectomy was performed within 4-6 weeks after completing NCRT/NICRT.

eAppendix 3. ComBat correction

The ComBat correction was first proposed for genomics to address the “batch effect” in microarray analysis, but it is also currently used in radiomics. In radiomics, the “batch effect” could be considered as the different image protocols, centers, machines, etc. [4]. The ComBat method is based on the the location and scale (L/S) family of corrections, which assumes that the errors introduced by batch differences can be adjusted by standardizing the means and variances across batches [4]. In radiomics, ComBat works by first standardizing the data. Subsequently, hyperparameters are estimated using the method of moments, which are then used to compute the empirical Bayes estimates of conditional posterior means feature-wise by center for the center effects parameters[1; 5].

References

1 Shi L, Li C, Bai Y et al (2025) CT radiomics to predict pathologic complete response after neoadjuvant immunotherapy plus chemoradiotherapy in locally advanced esophageal squamous cell carcinoma. Eur Radiol 35:1594-1604

2 van Hagen P, Hulshof MC, van Lanschot JJ et al (2012) Preoperative chemoradiotherapy for esophageal or junctional cancer. N Engl J Med 366:2074-2084

3 Li C, Zhao S, Zheng Y et al (2021) Preoperative pembrolizumab combined with chemoradiotherapy for oesophageal squamous cell carcinoma (PALACE-1). Eur J Cancer 144:232-241

4 Mahon RN, Ghita M, Hugo GD, Weiss E (2020) ComBat harmonization for radiomic features in independent phantom and lung cancer patient computed tomography datasets. Phys Med Biol 65:015010

5 Castaldo R, Brancato V, Cavaliere C et al (2022) A Framework of Analysis to Facilitate the Harmonization of Multicenter Radiomic Features in Prostate Cancer. J Clin Med 12

**Fig.S1** The flowchart of patients’ enrollment


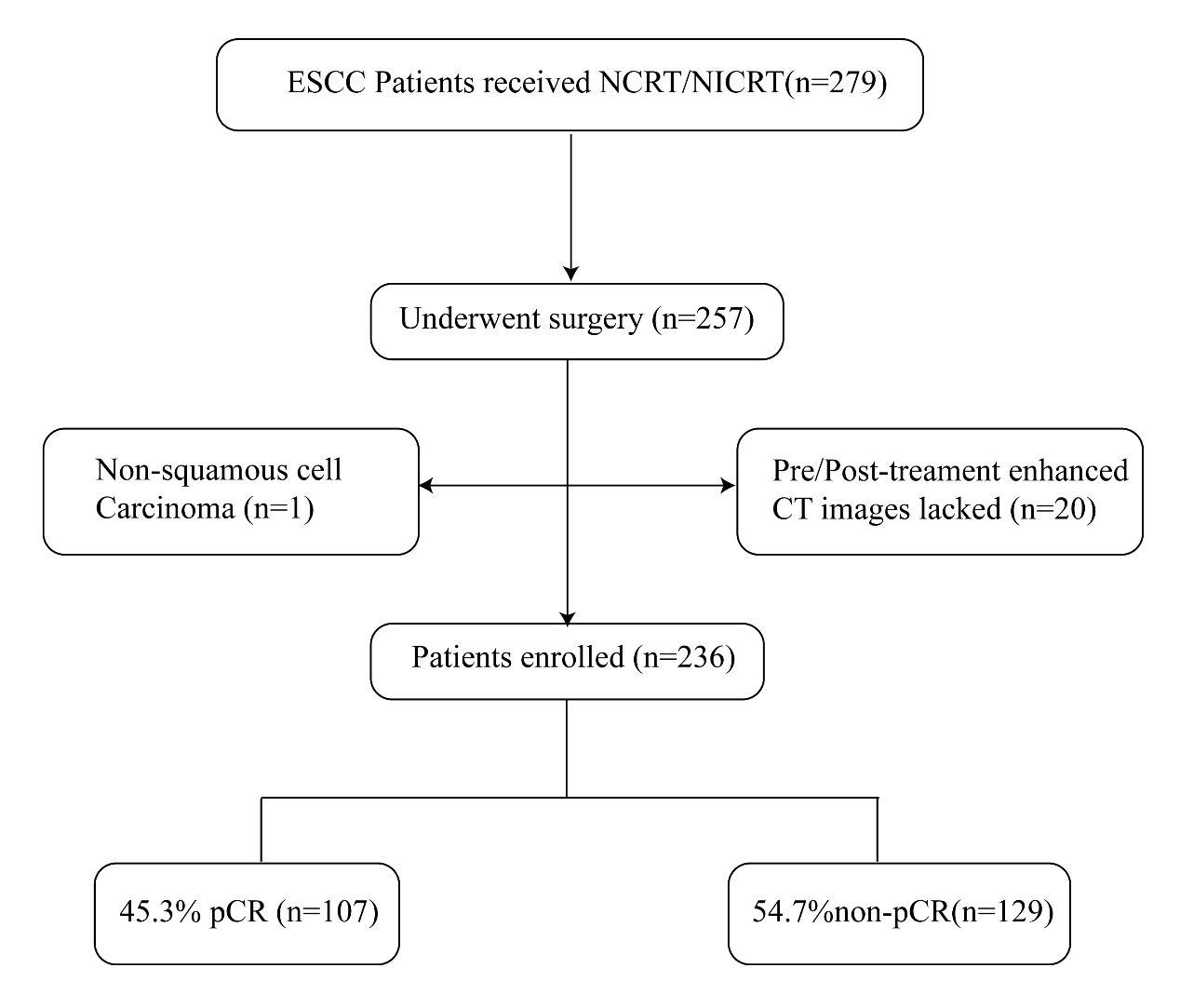


**Fig.S2** Comparison of AUC between radiomics model and clinical-radiomics model


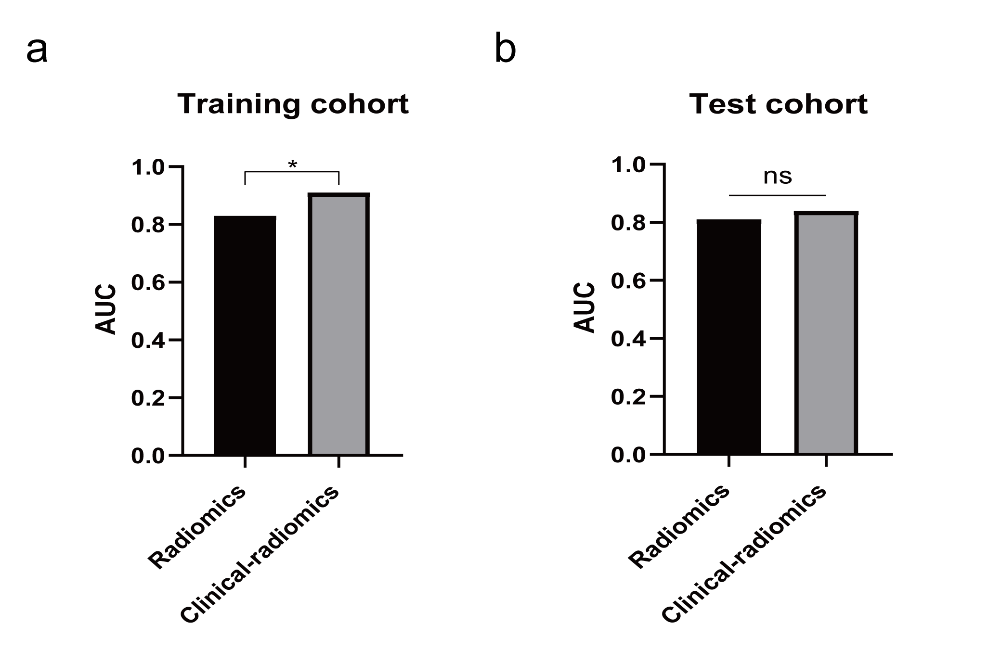

Supplement: ivag024_Supplementary_Data [file ivag024_supplementary_data.docx]
